# Chebulagic acid suppresses gastric cancer by inhibiting the AURKA/β-catenin/Wnt pathway

**DOI:** 10.3389/fphar.2023.1143427

**Published:** 2023-03-01

**Authors:** Jing Zhao, Yunfu Shi, Yubo Ma, Libin Pan, Yanan Wang, Li Yuan, Jinyun Dong, Jieer Ying

**Affiliations:** ^1^ Department of Gastric Surgery, The Cancer Hospital of the University of Chinese Academy of Sciences (Zhejiang Cancer Hospital), Hangzhou, China; ^2^ Institutes of Basic Medicine and Cancer (IBMC), Chinese Academy of Sciences, Hangzhou, China; ^3^ Zhejiang Provincial Research Center for Upper Gastrointestinal Tract Cancer, The Cancer Hospital of the University of Chinese Academy of Sciences (Zhejiang Cancer Hospital), Hangzhou, China; ^4^ Zhejiang Key Lab of Prevention, Diagnosis and Therapy of Upper Gastrointestinal Cancer, The Cancer Hospital of the University of Chinese Academy of Sciences (Zhejiang Cancer Hospital), Hangzhou, China; ^5^ Oncology Department, Tongde Hospital of Zhejiang Province, Hangzhou, China; ^6^ Key Laboratory of Cancer Prevention and Therapy Combining Traditional Chinese and Western Medicine of Zhejiang Province, Tongde Hospital of Zhejiang Province, Hangzhou, China; ^7^ Department of Hepato-Pancreato-Biliary and Gastric Medical Oncology, Cancer Hospital of the University of Chinese Academy of Sciences (Zhejiang Cancer Hospital), Hangzhou, China

**Keywords:** gastric cancer, Aurora A, chebulagic acid, biological functions, Wnt pathway

## Abstract

Gastric cancer (GC) is a prevalent malignant neoplasm that poses a serious threat to human health. Overexpression of Aurora A (AURKA) is frequently associated with the self-renewal and tumorigenicity of various cancers. Chebulagic acid (CA) has been examined as a potential tumor suppressor based on its ability against numerous tumor biological activities. However, the possible mechanisms of CA inhibition of the progression of GC by mediating the AURKA/β-catenin/Wnt signaling pathway have not been investigated. The present study investigated the level of AURKA expression in GC. We further examined the effect of CA on cell proliferation, migration, and apoptosis in the MKN1 and NUGC3 GC cell lines, and its efficacy in suppressing tumor growth was assessed in tumor bearing mice model. We demonstrated that AURKA was highly expressed in GC and associated with poor prognosis. We demonstrated that treatment with CA significantly inhibited the proliferation and migration of GC cells and induced apoptosis. Compared to the vehicle group, CA treatment severely diminished the volume and weight and the metastasis of tumors. CA also inhibited the expression of AURKA and the AURKA/β-catenin/Wnt signaling pathway *in vitro* and *in vivo*. Collectively, the present results demonstrated that high expression of AURKA may be an independent factor of poor prognosis in patients with GC, and CA significantly suppressed the tumor biological functions of GC and inhibited the AURKA/β-catenin/Wnt pathway.

## 1 Introduction

Gastric cancer (GC) is one of the most common malignant neoplasms, and it was diagnosed in more than 1 million people worldwide and resulted in more than 768,000 fatalities in 2020 (Ajani et al., 2022). It is also prevalent in China where it ranks as the second-highest morbidity and the third-leading cause of cancer-related fatalities, and it posed a severe risk to people’s lives and health (Arnold et al., 2020; Ajani et al., 2022). The best treatment option for early-stage GC is conventional surgery, but curative resections may result in tumor relapses and metastasis, which worsen patient’s prognosis (Liu et al., 2019; Liu et al., 2022b). Patients with advanced GC commonly receive first-line chemotherapy, radiation, immunotherapy, and targeted treatments. However, the therapeutic outcomes are typically restricted due to several unfavorable side effects that are unavoidable and cannot be disregarded (Thrift and El-Serag, 2020; Joshi and Badgwell, 2021). Unfortunately, patients with advanced GC still have an extremely low 5-year survival rate of approximately 10% (Chen et al., 2019). Therefore, it is urgent to elucidate the biological mechanisms of GC and identify a more effective treatment strategy.

Aurora A (AURKA) belongs to the aurora kinase family, and it regulates the cell cycle and dynamics of the centrosome and mitotic spindle. It is crucial for preserving the accuracy of genetic information (Hong et al., 2022; Liu et al., 2022a; Shi et al., 2022). Numerous malignancies, including GC, are associated with frequent amplification and/or overexpression of AURKA, and its levels are higher in most tumor tissues compared to adjacent normal tissues (Mou et al., 2021). The overexpression of AURKA is more commonly linked to stemness, chemotherapeutic resistance, mesenchymal phenotype, and cell proliferation, and it is an important independent prognostic factor in human malignancies (Bao et al., 2022; Li et al., 2022b; Chan et al., 2023). The role of AURKA as an oncogenic driver in the regulation of numerous molecules and signaling pathways has been investigated (Lin et al., 2020). Emerging studies demonstrated that several AURKA substrates, including GSK3β and β-catenin, are also involved in crucial carcinogenic signaling as well (Zou et al., 2022). AURKA and GSK3β form a complex in GC cells. The overexpression of AURKA significantly promotes GSK3β phosphorylation at the Ser9 site, which stabilizes β-catenin levels and activates Wnt signaling (Dutta-Simmons et al., 2009; Bornschein et al., 2016). AURKA overexpression was implicated in the upregulation of β-catenin expression and led to an increase in β-catenin stability by blocking ubiquitin-mediated proteasomal degradation and suppressing phosphorylated β-catenin at the Ser552 and Ser675 sites (Jin et al., 2015; Jacobsen et al., 2018). Stabilized β-catenin moves from cell-cell interactions to the nucleus and triggers the transcription of target genes (Zhang and Wang, 2020). Disruption of these related biological pathways prevents tumorigenesis and development.

Natural products are the main source for the prevention and treatment of human disease because these products contain a wide variety of compounds with unique structures (Wang et al., 2021). *Terminalia chebula* is a member of the Combretaceae family that is native to Southeast Asia, and its pharmacological properties are under investigation (Kumar et al., 2014). The fruit powder of *T. chebula* has been used as medicine since ancient times for the treatment of digestive, infectious, and allergic diseases (Athira et al., 2017). Nowadays, the water or ethanolic extracts of powder has also been used to cure cancer and other oxidative stress-related disorders (Ferrucci et al., 2016; Kim et al., 2022). Chebulagic acid (CA) is a benzopyran tannin and major constituent in the fruits of *T. chebula*, and it has strong anti-angiogenic, anti-inflammatory, anti-virus, and anticancer effects (Lin et al., 2018; Shanmuganathan and Angayarkanni, 2018). However, the molecular mechanisms of CA in GC are still unclear.

The present study demonstrated that CA exerted an antitumor effect *in vivo* and *in vitro*. CA suppressed proliferation, migration, and clonogenicity, and induced apoptosis of MKN1 and NUGC3 cell lines, and slowed the growth of the tumor with few side effects and high efficacy in tumor bearing nude mice. Therefore, we further examined that CA inhibited the progression of GC by regulating the AURKA/β-catenin/Wnt signaling pathway, which exhibited its potential in antitumor.

## 2 Materials and methods

### 2.1 Tissue microarray construction

GC tumor tissue and matched paracancerous tissues from 160 GC patients were collected from the Zhejiang Cancer Hospital between January 2013 and December 2017. Patients had not received radiation, immunotherapy, or chemotherapy prior to specimen collection. All available clinicopathological factors and complete survival follow-up information were assessed. Informed consent was obtained from all patients before testing. Tissue microarrays were constructed (Yuan et al., 2021). Briefly, brown-stained cells were regarded as positive after 2 expert pathologists independently assessed the slides without knowledge of the clinical outcomes. The intensity of AURKA expression was classified into 4 groups using the H-score system: 0, 3, 6, and 12. A score of 0 indicated no staining, and weak, moderate, and intense staining were indicated by scores of 3 (1%–25%), 6 (26%–50%), and 12 (more than 50%), respectively.

### 2.2 Cell culture and chemicals

The human GC cell lines MKN1, NUGC3, and normal gastric epithelial cell line GES-1 were purchased from the cell bank of the Chinese Academy of Science (Shanghai, China). All cells were maintained in RPMI 1640 medium containing 10% FBS, 100 U/mL penicillin, and 100 μg/mL streptomycin and incubated in a humidified atmosphere at 37°C in 5% CO_2_.

CA (Cat# TQ0180) was obtained from TargetMol (Boston, MA, United States of America). Primary antibodies against AXIN2 (Cat#20540-1-AP), p-β-catenin (Cat#28853-1-AP), β-catenin (Cat#51067-2-AP), p-GSK3β (Cat#67558-1-Ig), GSK3β (Cat#51065-1-AP), and GAPDH (Cat#60004-1-Ig) were purchased from Proteintech (Wuhan, China). Anti-AURKA (Cat#91590S) antibodies were obtained from Cell Signal Technology (Danvers, MA, United States of America).

### 2.3 Cell viability assay

MKN1, NUGC3, and GES-1 cells were seeded into 96-well microplates at a density of 5 × 10^3^ cells/well and cultured overnight. Different CA concentrations ranging from 0 to 128 μM were incubated with cells for an additional 48 h. Next, 100 μl of a solution (CCK8 assay kit: medium = 1:10) was incubated for approximately 4 h, and cell proliferation was detected at a wavelength of 450 nm.

### 2.4 Transwell migration assay

After digestion, MKN1 and NUGC3 cells were resuspended in serum-free medium at an adjusted cell density of 3.5 × 10^5^ cells/ml. The upper and bottom Transwell chambers (Corning, NY, United States of America) were filled with 200 μL of cell suspension and 700 μl of medium with 10% FBS, respectively. After 48 h of CA treatment (0, 3, 6, 12 μM for MKN1 cells and 0, 5, 10, 20 μM for NUGC3 cells), the culture plate was removed from the incubator. The cells in the upper chamber were removed with cotton swabs. Crystal violet was used to stain the cells after fixation in 4% paraformaldehyde in the lower chamber. Cells in the lower chamber were photographed and counted. The migrating cells were calculated using ImageJ software.

### 2.5 Wound healing assay

A culture-insert 4-well chamber (Ibidi, Germany) was used in the wound healing assay, and 7 × 10^4^ MKN1 or NUGC3 cells were seeded onto each well and incubated overnight. The inserts were lifted with sterile tweezers, and the cell area beneath the inserts was washed 3 times to remove any floating cells. The cells were maintained in RPMI 1640 containing 1% FBS with different concentrations of CA (0, 3, 6, 12 M for MKN1 cells and 0, 5, 10, 20 M for NUGC3 cells). An inverted microscope (IX71, Olympus, Japan) was used to capture images of the cell migration process into the wound space at 0 and 48 h. The rate of wound healing was calculated using ImageJ software.

### 2.6 Colony formation assay

The proliferation of GC cells was examined using the colony formation assay. Briefly, the cells were seeded at 1000 cells/well in a single cell layer in 6-well plates, and CA treatment was applied for 48 h (0, 3, 6, 12 μM for MKN1 cells and 0, 5, 10, 20 μM for NUGC3 cells). The cells were cultured in CA-free complete medium for an additional 10 days, and the medium was refreshed every 3 days. The colonies were stained with crystal violet, and colonies larger than 200 μm were counted using ImageJ software.

### 2.7 Flow cytometry analysis

The Annexin V-FITC Apoptosis Detection Kit (Beyotime, Shanghai, China) was used to detect the cell apoptosis rate. Briefly, human GC cell lines (MKN1 and NUGC3) were plated in 6-well culture plates at 3 × 10^5^ cells per well and incubated overnight, followed by treatment with CA (0, 3, 6, 12 μM for MKN1 cells and 0, 5, 10, 20 μM for NUGC3 cells) for 48 h. The cells were washed twice with PBS and resuspended in 200 μl binding buffer. Annexin V-FITC (5 μl) and PI (10 μl) were added into resuspended cells. After 15 min of incubation at room temperature in the dark, the samples were analyzed using flow cytometry (FACSCalibur, United States of America). Analysis was performed using FlowJo software.

### 2.8 Tumor bearing mice model

To establish the subcutaneous GC mice model, the left axilla of the recipient mouse was subcutaneously injected with 5 × 10^5^ MKN1 cells. After the subcutaneous GC mice model was successfully established, tumor bearing mice were randomly assigned to vehicle and treatment groups (5 mg/kg and 10 mg/kg CA). Tumor volumes were assessed using a Vernier caliper every 5 days and calculated using the formula length × width × width × 0.5. Mice’s body weights, health state, and tumor sizes were monitored. Based on the tumor volume, the tumor inhibition rate was determined. The nude mice were euthanized after the 30-day intervention, and the tumor tissue and organs were collected and stored in a freezer at −80°C for further use.

Orthotopic GC mice model was established as previously reported (Zhang et al., 2017). Briefly, 5 × 10^6^ MKN1-Luc cells were subcutaneously injected into the left axilla of a nude mouse. When the tumor volume reached approximately 500 mm^3^, the tumor was removed sterilely and minced into 1–2 mm^3^ fragments with scissors. One fragment was immobilized onto the stomach surface of nude mice *via* surgery. One week after surgery, IVIS Lumina LT (Caliper Life Sciences, United States of America) was used to assess the intensity of orthotopic tumors in nude mice 10 min after the injection of D-luciferin sodium salt (150 mg/kg). Positive fluorescence intensity indicated successful establishment of the orthotopic GC mice model. The orthotopic GC mice model was randomly divided into the vehicle and CA (5 mg/kg and 10 mg/kg) groups according to the fluorescence intensity and treated for 4 weeks. Mice body weights, health state, and tumor sizes were monitored. The fluorescence data were acquired and analyzed by Living Image Ver. 4.3 (Caliper Life Sciences, United States of America) software.

### 2.9 Immunohistochemical (IHC) staining

Tumor tissues from 160 GC patients and GC mice model were removed, fixed in 10% formaldehyde and embedded in paraffin. The tissues were continuously sliced, dewaxed, rehydrated in an alcohol series, and subjected to antigen. Goat serum (10%) was used to block the sections for 30 min at 37°C. The slides were incubated with primary antibodies overnight at 4°C followed by a 30 min incubation with secondary antibodies. Staining was visualized using DAB for 3 min.

### 2.10 Western blotting

Cells were lysed for 15 min in RIPA lysis buffer (Beyotime, Shanghai, China) containing a proteinase inhibitor cocktail (TargetMol, United States of America) after treatment with CA for 48 h. A Bradford assay kit (Beyotime, Shanghai, China) was used to quantify and homogenize the whole-cell lysates. Proteins were separated using SDS-PAGE gels and transferred onto PVDF membranes (Millipore, United States of America). Followed by incubation with primary antibodies (AXIN2, 1:1000; p-β-catenin,1:2000; β-catenin,1:5000; p-GSK3β, 1:1000; GSK3β, 1:1000; AURKA, 1:1000; GAPDH, 1:2000) at 4°C overnight. After incubation with the secondary antibody (1:5000) at 37°C for 1 h, enhanced chemiluminescence (ECL) was used to detect the signals and GAPDH levels were used to confirm that the proteins had loaded equally.

### 2.11 Statistical analysis

All data were analyzed using GraphPad Prism (Graph Pad Software, United States of America) and expressed as the means ± SD. Differences between groups were analyzed using the one-way ANOVA test. Survival curves were estimated using the Kaplan-Meier method and log-rank test. A *p*-value <0.05 was considered statistically significant.

## 3 Results

### 3.1 AURKA was overexpressed in GC tissue and associated with poor prognosis of GC patients

**FIGURE 1:**
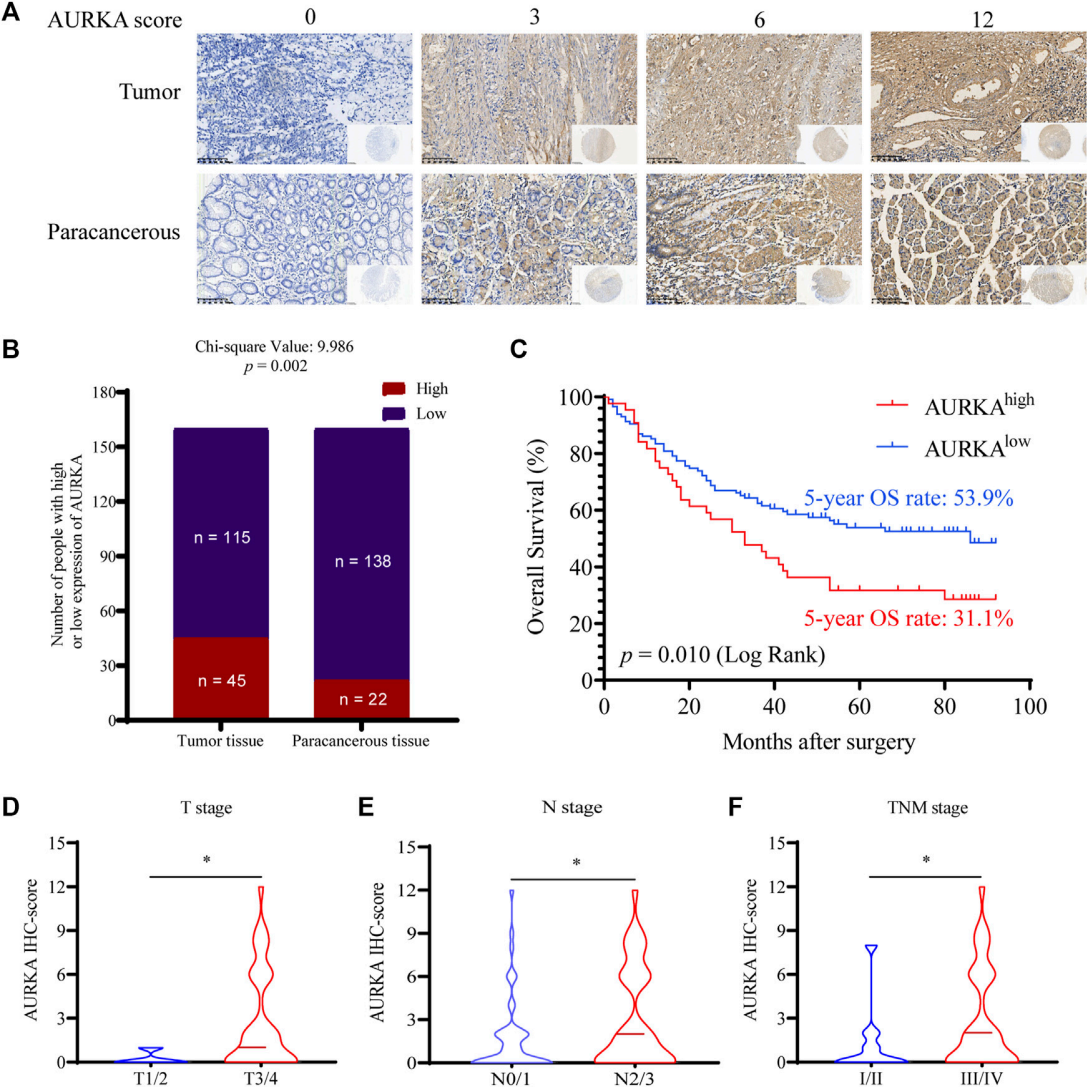
AURKA is highly expressed in GC tissues and is associated with poor prognosis in GC. **(A)** Representative images of IHC staining of AURKA with different H-scores in 160 GC patient tissue microarray constructions (400×). **(B)** Differential expression of AURKA in GC tissue and matched adjacent tissues (*p* = 0.002). **(C)** The survival curves of GC patients with different AURKA expression levels (*p* = 0.01). **(D)** The AURKA IHC score in GC patients with different T stages, *p < 0.05. **(E)** The AURKA IHC score in GC patients with different T stages, *p < 0.05. **(F)** The AURKA IHC score in GC patients with different T stages, *p < 0.05.

To investigate the level of AURKA expression in GC tissues and its clinical significance, tissue microarrays from 160 patients with GC were examined using IHC staining and evaluated by the H-score system (Figure 1A). AURKA (scores 6 and 12) was overexpressed in tumor tissues of 45 (28.13%) GC cases, and the other 115 (71.87%) cases exhibited low expression. However, there were only 22 (13.75%) GC cases with intense AURKA staining, and 138 (86.25%) cases exhibited weak staining in paracancerous tissues (*p* = 0.002), which suggested that high expression of AURKA was associated with GC (Figure 1B). The relationship between the level of AURKA expression in GC tissues and overall survival was investigated. The 5-year OS rate was 53.9% in GC patients with low AURKA expression compared to 31.1% in patients with high expression (*p* = 0.01), which demonstrated that the high expression of AURKA in GC tissues correlated with a shorter survival time (Figure 1C). We also examined the relationship between the clinicopathological features of GC patients and the level of AURKA expression (Table 1). Correlation analysis of AURKA expression revealed no significant difference in age, sex, or tumor size between the low and high AURKA expression groups. The high AURKA expression group had more T3/T4 patients (*p* = 0.033, Figure 1D) and more N2/N3 patients (*p* = 0.005, Figure 1E). For the TNM stage, the high AURKA expression group had more stage III and stage IV patients than the low AURKA expression group (*p* = 0.041, Figure 1F). These results showed that AURKA was highly expressed in GC and correlated with poor prognosis.

**TABLE 1:** Relationship between AURKA expression and clinicopathological variables in 160 GC patients.

Variables	Expression of AURKA		N	χ2	*p*-Value
	High	Low			
*Sex*					
Male	31	83	114	0.170	0.680
Female	14	32	46		
*Age (years)*					
<60	15	50	65	1.138	0.240
≥60	30	65	95		
*Tumor size (cm)*					
<5	10	42	52	2.981	0.084
≥5	34	71	105		
*Tumor location*					
Distal stomach	22	77	99	6.359	0.042*
Proximal stomach	17	33	50		
Total stomach	6	5	11		
*T stage*					
T1+T2+T3	2	20	22	4.571	0.033*
T4	43	95	138		
*N stage*					
N0+N1	6	40	46	7.763	0.005*
N2+N3	39	72	111		
*TNM stage*					
I+II	2	19	21	4.137	0.041*
III+IV	43	96	139		
*AFP (ng/ml)*					
≤10	38	111	149	9.221	0.002*
>10	6	2	8		
*CEA (ng/ml)*					
≤5	29	88	117	2.401	0.121
>5	16	27	43		
*CA19-9 (U/ml)*					
≤37	29	76	105	0.001	0.983
>37	15	39	54		
*CA72-4 (U/ml)*					
≤6.9	25	86	111	4.660	0.031*
>6.9	13	18	31		
*CA125 (U/ml)*					
≤35	39	107	146	0.823	0.364
>35	5	8	13		

* Statistically significant (*p* < 0.05).

### 3.2 CA inhibited proliferation, migration, and induced apoptosis of GC cells

**FIGURE 2:**
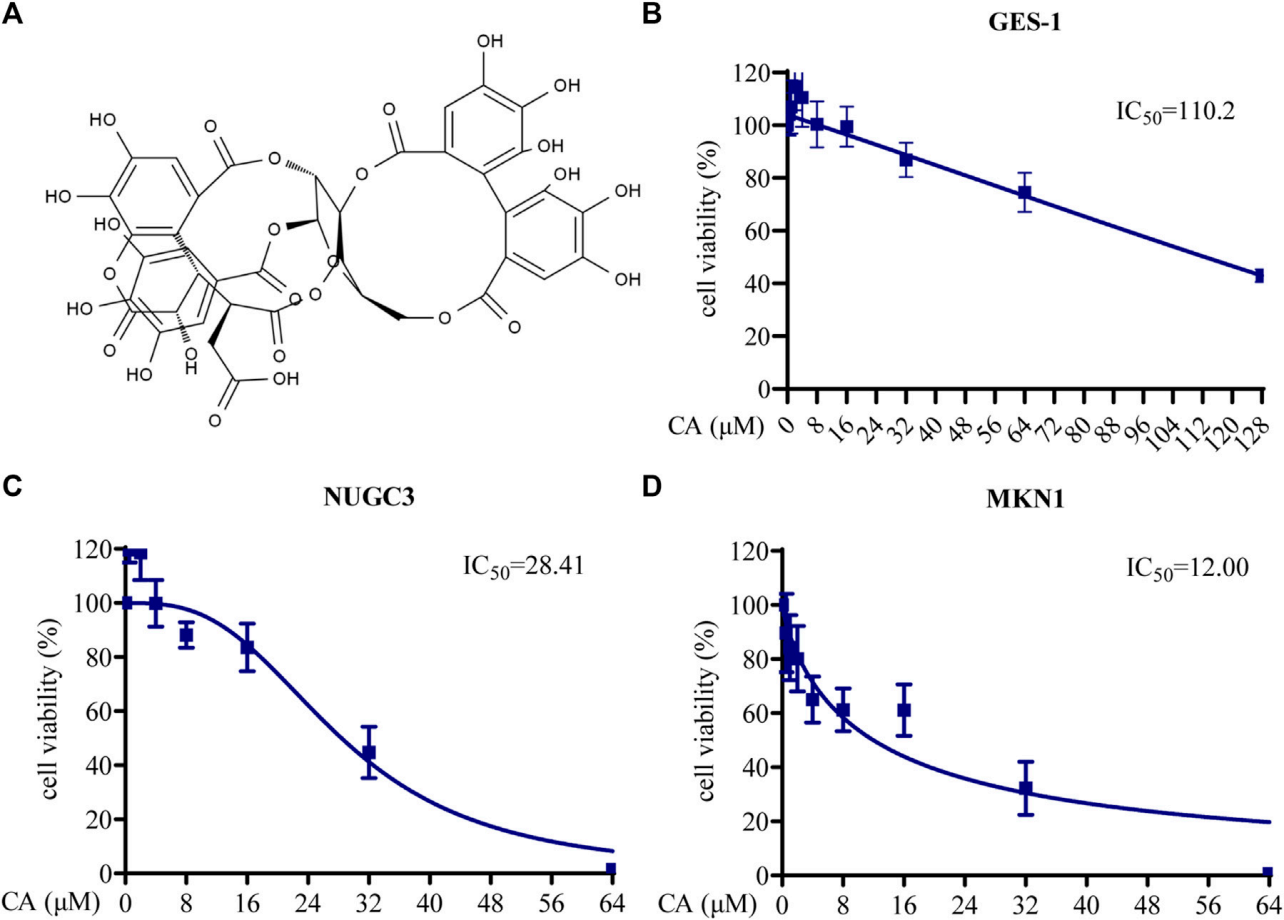
The cytotoxicity of CA in GC cells. **(A)** The structural formula of CA. **(B)** Cell viability of GES-1 cells treated with different concentrations of CA. **(C)** Cell viability of NUGC3 cells treated with different concentrations of CA. **(D)** Cell viability of MKN1 cells treated with different concentrations of CA.

The structure of CA is shown in Figure 2A. To assess the biological functions of CA in GC cells, CCK8, Transwell migration, wound healing, colony formation, and apoptosis assays were used. The cytotoxicity of CA was examined using a CCK8 assay with MKN1, NUGC3, and GES-1 cells treated with different concentrations (0–128 μM) of CA for 48 h. The IC_50_ values indicated that CA had a 50% inhibitory effect on GES-1, MKN1, and NUGC3 cells at 110.20 μM, 12.00 μM, and 28.41 μM, respectively (Figures 2B–D). GES-1 cells exhibited a much higher IC_50_ value than the GC cell lines, which suggested that CA had selective cytotoxicity towards GC cells.

**FIGURE 3:**
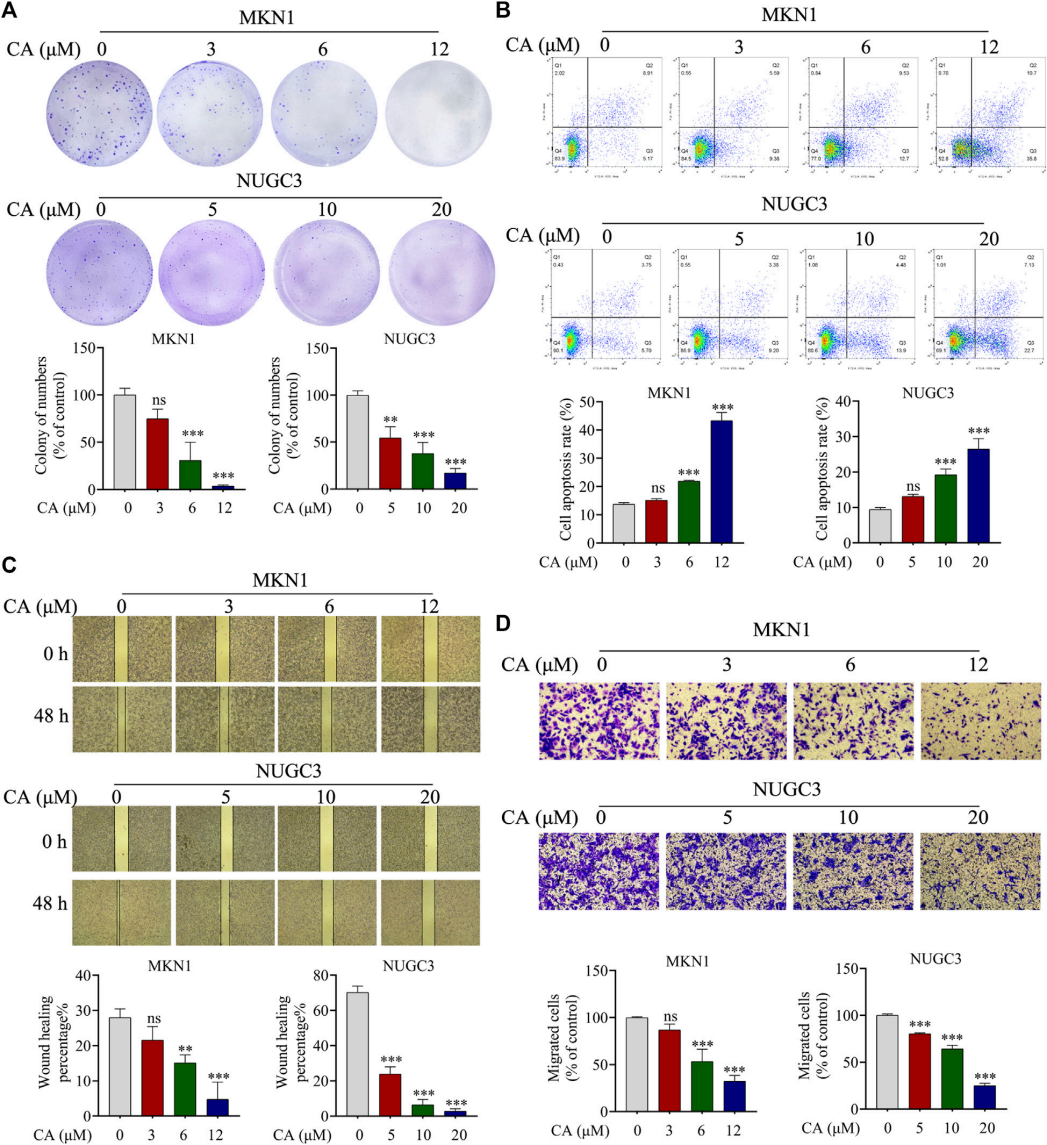
CA inhibition of biological functions in GC cells. **(A)** Colony formation assays for MKN1 and NUGC3 cells were performed following treatment with CA. **(B)** A wound healing assay was performed following CA treatment. **(C)** Transwell migration assays were performed following CA treatment. **(D)** Induction of apoptosis of MKN1 and NUGC3 cells after CA treatment. **p < 0.01, ***p < 0.001, ns *p* > 0.05 compared to the control group (n = 3). One-way ANOVA test. All data are shown as the means ± SD.

The colony formation assay was used to evaluate the proliferation ability of MKN1 and NUGC3 cells following treatment with different concentrations of CA. As shown in Figure 3A, CA hindered the number of cell colonies formed in a dose-dependent manner in GC cells. Annexin V-FITC/PI flow cytometric analysis indicated that the ratio in the early and late stages of apoptosis was markedly enhanced in the groups treated with different concentrations of CA compared to the control groups, which suggested that CA triggered the cellular apoptosis of GC cells (Figure 3B). We focused on the impact of CA treatment on GC cell migration. As expected, the wound healing assay results showed that GC cells treated with CA exhibited a slower wound healing rate than the control group (Figure 3C). These findings were also supported by the Transwell assay. CA treatment significantly reduced the number of migrating cells and inhibited cell migration (Figure 3D). These results indicated that CA treatment influenced the biological function of GC cells.

### 3.3 CA suppressed GC growth and metastasis *in vivo*

**FIGURE 4:**
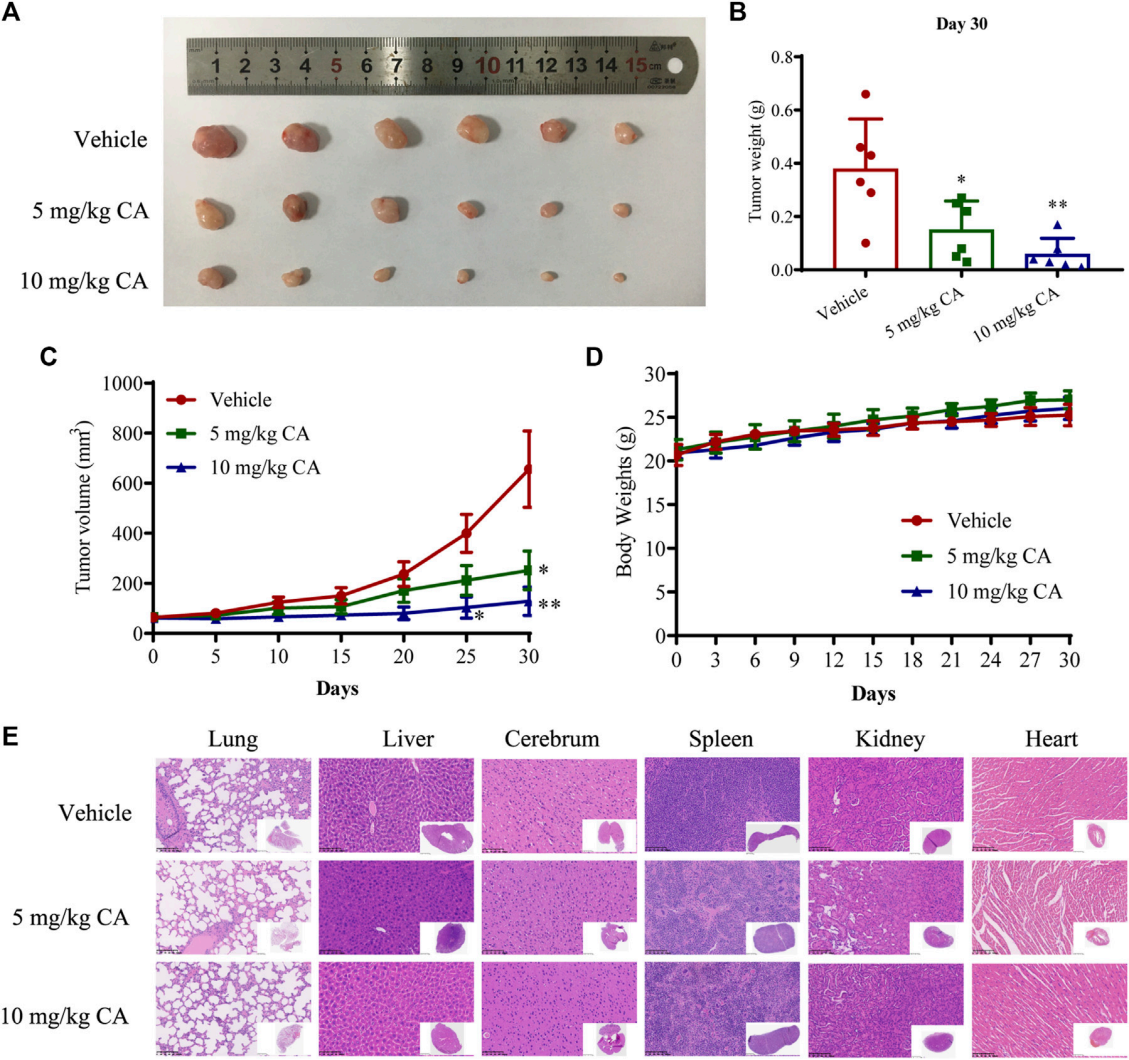
Inhibition effect of CA on tumor growth of GC in MKN1 xenograft mice models. **(A)** Tumor image of MKN1 xenograft mice after 30 days of treatment with vehicle or CA (5 mg/kg and 10 mg/kg). **(B)** Tumor weight after 30 days of treatment with vehicle or CA (5 mg/kg and 10 mg/kg). **(C)** The tumor volume curves of mice during 30 days. **(D)** Average body weight of mice during 30 days. **(E)** Representative H&E staining images of the heart, liver, spleen, lung, kidney, and cerebrum of mice (400×). *p < 0.05, **p < 0.01 compared to the vehicle group (n = 6). One-way ANOVA test. All data are shown as the means ± SD.

To verify the inhibitory effects of CA on the growth and metastasis of GC *in vivo*, we examined the curative efficacy of CA in MKN1 xenograft and orthotopic mice models. MKN1 xenograft mice were treated with CA by intragastric administration at 5 and 10 mg/kg/day for 30 days. The tumor tissues were removed for photographing and weighing after the mice were sacrificed. Compared to the vehicle group, the tumor sizes and weights were significantly decreased with CA treatment at 5 and 10 mg/kg (Figures 4A, B). Every 5 days during the tumor growth phase, the tumor volumes were recorded. These results demonstrated that CA treatment continuously inhibited tumor volumes over time (Figure 4C). Body weights of the mice were monitored every 3 days, but no marked variations were found between these 3 groups (Figure 4D). Little tissue damage was observed in the H&E staining of organs, such as the heart, liver, spleen, lung, kidney, and cerebrum (Figure 4E).

**FIGURE 5:**
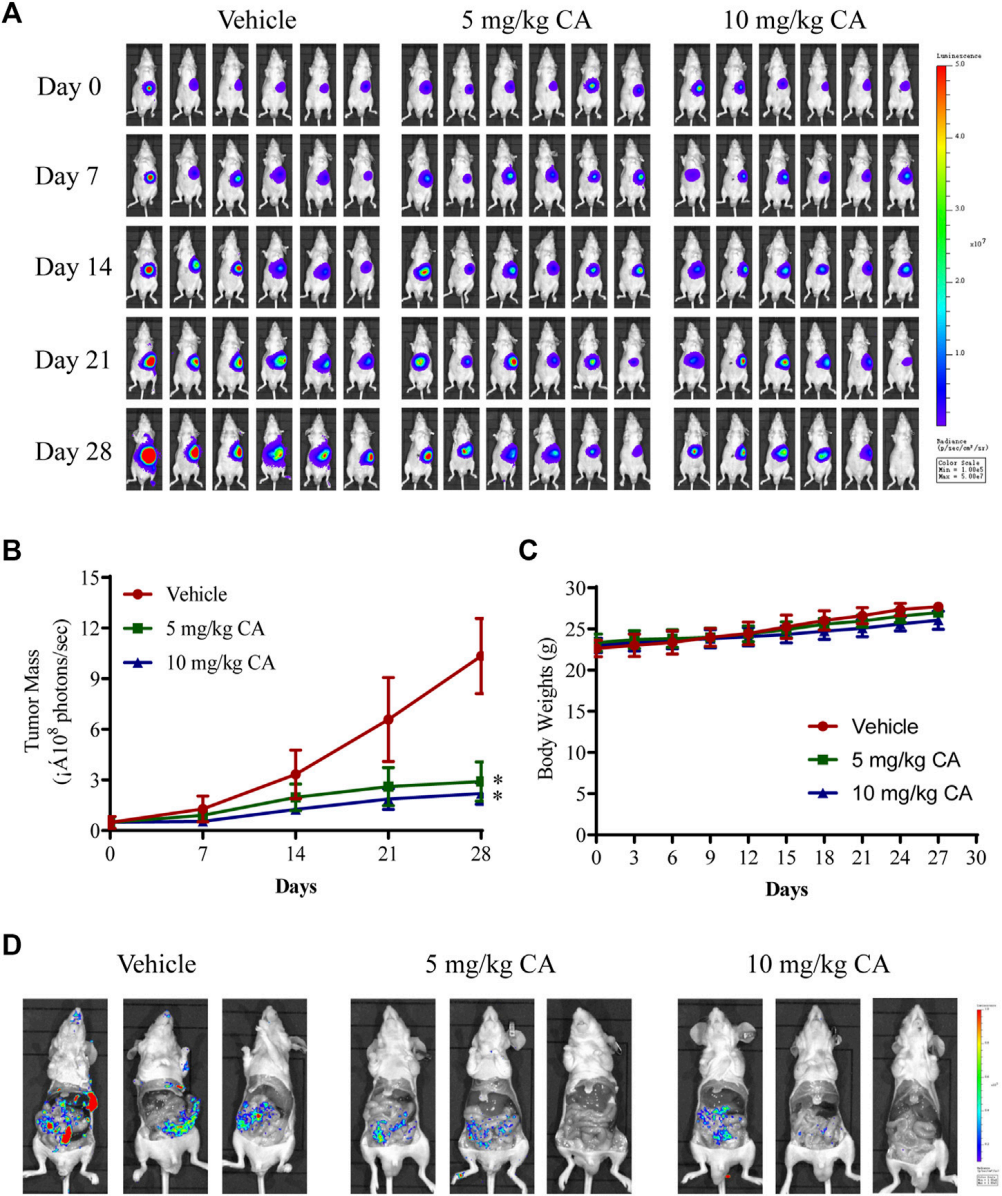
Inhibition effect of CA on tumor growth and metastasis of GC in MKN1 orthotopic mice models. **(A)** Fluorescence images of the tumor were detected every 7 days for 4 weeks. **(B)** The tumor mass curves of mice during 4 weeks. **(C)** Average body weight of mice during 4 weeks. **(D)** Representative fluorescence images of peritoneal metastasis of tumor. *p < 0.05 compared to the vehicle group (n = 6). One-way ANOVA test. All data are shown as the means ± SD.

We further evaluated the antitumor and antimetastatic effects of CA in MKN1 orthotopic mice. The fluorescence intensity of tumors was monitored every 7 days during tumor growth, and similar findings were observed. CA treatment (5 and 10 mg/kg) markedly slowed the growth of MKN1 orthotopic tumors (Figures 5A, B), but there was no significant difference between the 5 and 10 mg/kg groups. Treatment with CA had no significant impact on body weights compared with the vehicle group in MKN1 orthotopic mice (Figure 5C). We also detected the fluorescence intensity in the peritoneum of mice after removal of the tumor tissue, and the results showed that the intensity of the vehicle group was more obvious than the CA treatment groups, which demonstrated the ability of CA to inhibit tumor metastasis (Figure 5D).

### 3.4 CA inhibited the AURKA/β-catenin/Wnt signaling pathway *in vivo* and *in vitro*

**FIGURE 6:**
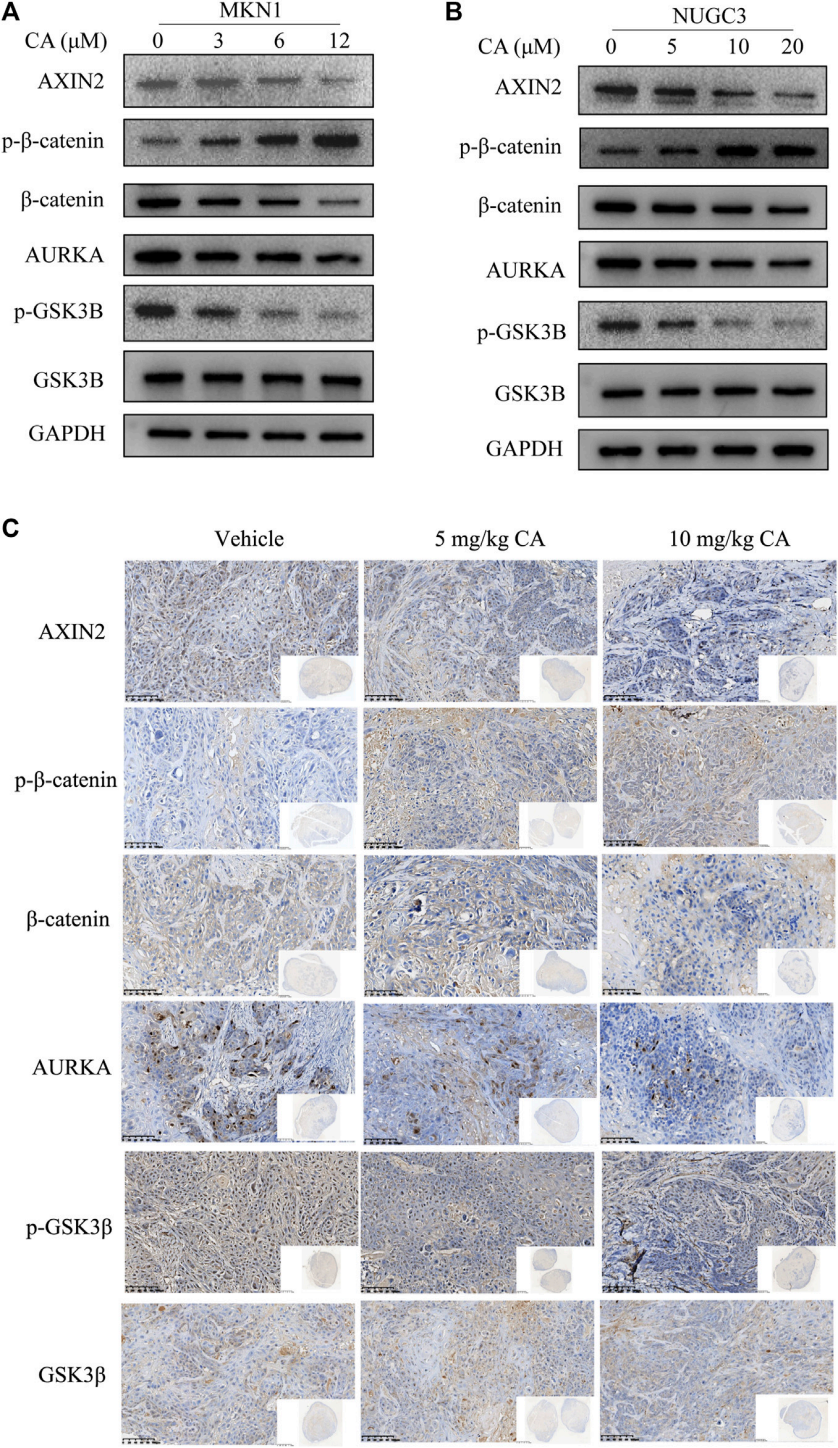
CA inhibits the AURKA/β-catenin/Wnt pathway *in vitro* and *in vivo*. **(A)** Western blotting analysis of the indicated proteins in MKN1 cells treated with different concentrations of CA for 48 h. **(B)** Western blotting analysis of the indicated proteins in NUGC3 cells treated with different concentrations of CA for 48 h **(C)** IHC images of the indicated proteins in randomly selected MKN1 xenograft tumor sections after 30 days of treatment with different dosages of CA ( × 400).

AURKA induces the progression of tumors by participating in cancer cell metastasis, apoptosis, growth, epithelial-mesenchymal transition, and the self-renewal of cancer stem cells (Du et al., 2021). We also observed that AURKA was highly expressed in GC tissues. Several studies reported that some AURKA substrates, including GSK3β and β-catenin, participated in crucial oncogenic signaling (Mou et al., 2021), and AURKA directly phosphorylated GSK3β in cancer cells, which stabilized β-catenin and activated Wnt signaling (Dar et al., 2009). To examine whether CA treatment reversed this mechanism in GC cells, we examined the expression of the AURKA/β-catenin/Wnt signaling pathway using Western blotting and IHC staining. We first measured the impact of CA on AURKA in MKN1 and NUGC3 cells. Our results showed that CA markedly suppressed the level of AURKA expression, and p-GSK3β (Ser9) was also significantly reduced with the total GSK3β level remaining unchanged. We detected a significant decrease in the AXIN2 and β-catenin protein levels and a great accumulation in p-β-catenin in MKN1 and NUGC3 cells (Figures 6A, B). Our *in vivo* study revealed the same efficacy in AURKA/β-catenin/Wnt as the *in vitro* studies (Figure 6C).

## 4 Discussion

GC is the second most frequently diagnosed cancer, and it accounts for 10% of all cancer-related fatalities globally (Lordick et al., 2022). Despite advancements in treatment, individuals with GC have a poor 22% 5-year OS rate (Li et al., 2022a). Therefore, the discovery of effective therapeutics is crucial to overcome this problem. Therefore, we verified the role of AURKA in GC and examined the beneficial effect of CA against GC by inhibiting the AURKA/β-catenin/Wnt signaling pathway.

AURKA is a key cell cycle regulator located on the 20q13 chromosomal region (Jacobsen et al., 2018). Gain of 20q is associated with a poor prognosis in many cancer types (Zhang et al., 2021). The TCGA database also states that high expression of AURKA is a prognostic marker in several cancers (Du et al., 2021). The inhibition of AURKA may have considerable antitumor effects, but the ability was only demonstrated in predicting patient survival in kidney renal clear cell carcinoma, brain lower-grade glioma, and kidney renal clear cell carcinoma. We examined the association between the expression of AURKA in GC tissues and the corresponding paracancerous tissues and the overall survival in 160 cases. We demonstrated that high expression of AURKA positively correlated with poorer overall survival in GC patients, which indicated that AURKA was associated with GC development and advanced clinical stage. High levels of AURKA expression in patients predicted a poor prognosis and were closely associated with T, M, and TNM stages (*p* < 0.05).

Nowadays, medicinal herbs have been used as one of the favorites for the preparations of numerous novel anticancer compounds (Rameshk et al., 2018; Mehrabani et al., 2020). More than 60% of available chemotherapeutic medications come from a variety of natural sources, including plants, microbes, and marine species. Accumulating evidence suggests that natural medicines are crucial in the prevention and treatment of tumors as a source of novel bioactive chemicals. CA is sourced from the fruits of *T. chebula*, and it has been used in the treatment of several cancers. CA have also exanimated inhibition of GSK3β-dependent serine phosphorylation of β-catenin (Athira et al., 2017). The link between AURKA and Wnt signaling is not a novel finding, p-GSK3B and AXIN2 are often deregulated by AURKA, which leads to stimulation of Wnt signaling in GC cell lines. Previous studies reported that AURKA overexpression disrupted the β-catenin destruction complex by competing with AXIN2, which subsequently reduced the p-β-catenin level and stabilized the total β-catenin. The stabilized β-catenin enters the nucleus and activates Wnt signaling (Liu et al., 2022b). The present results support the potential anticancer ability of CA and its impact on the AURKA/β-catenin/Wnt signaling pathway. However, the effects of CA on the AURKA/β-catenin/Wnt signaling pathway in GC have not been reported, and whether CA exerts its antitumor function in GC remains unclear, and requires clarification. The AURKA/β-catenin/Wnt signaling pathway plays important roles in regulating various biological processes, including cell proliferation, apoptosis, invasion, and tumorigenesis (Duda et al., 2020). Based on this activity, we verified the effects of CA in inducing antitumor activities and the impact of the AURKA/β-catenin/Wnt signaling pathway.

The inhibition of uncontrolled cell proliferation is crucial to hindering tumor progression (Xie et al., 2021). The present study revealed that CA inhibited the viability of GC cell lines (MKN1 and NUGC3) in a concentration-dependent manner. Based on these findings, a suitable CA concentration was used in subsequent experiments to evaluate the effects of CA on cellular biological behaviors. The results revealed that treatment with CA significantly reduced cell colony formation, which indicated that CA exerted a suppressive effect on cell proliferation. Furthermore, tumor migration is one cause of tumor recurrence, and our results showed that CA inhibited GC cell migration *in vitro*. Besides, apoptosis maintains homeostasis of the internal environment under physiological conditions, and an imbalance between cell division and cell death is often induced in cancer cells (Yi et al., 2018). Therefore, inducing apoptosis remains the main method and the marker of chemotherapy success. Our Annexin-FITC/PI results demonstrated that CA induced apoptosis of GC cells in a concentration-dependent manner. To further investigate the roles of CA in the progression of GC, we established two different models, such as the MKN1 xenograft and orthotopic mice models, to investigate the antitumor effect of CA *in vivo*. After intragastric CA administration, tumor weights and volumes of the CA-treated group were markedly decreased without causing any host toxicity, which suggested that CA inhibited tumor growth *in vivo*. Furthermore, tumor metastasis often occurs with GC development and is highly associated with death (Zhu et al., 2018). CA treatment inhibited the peritoneum metastasis of MKN1 orthotopic mice models in the present study. Our results showed that CA had marked markedly anti-GC effects *in vitro* and *in vivo*.

AURKA interacts with GSK3β and directly binds to and phosphorylates GSK3β at Ser9 (Jin et al., 2015; Liu et al., 2022b). GSK3β is a major protein kinase that regulates the phosphorylation of β-catenin in the cytoplasm and targets it for degradation (Law and Zheng, 2022). The inactivation of GSK3β by phosphorylation reduces the ubiquitination of β-catenin, which results in the nuclear accumulation and transcriptional activity of β-catenin (Guo et al., 2022). We detected a decrease in the level of AURKA following treatment with CA at different concentrations, which corresponded to the inhibition of p-GSK3β, AXIN2, and β-catenin with a significant upregulation in the phosphorylation level of β-catenin protein levels in GC cells and tumor of MKN1 xenograft models. These results suggested that CA treatment downregulated the AURKA/β-catenin/Wnt signaling pathway.

In conclusion, we found that the expression of AURKA highly positively correlated with poor prognosis in GC patients. The present study determined for the first time, to the best of our knowledge, that CA inhibited GC cell biological functions *in vitro* and inhibited tumor growth *in vivo*. CA exerted its antitumor effects partially by inhibiting the AURKA/β-catenin/Wnt signaling pathway. These findings provide a promising strategy for the treatment of GC.

## Data Availability

The original contributions presented in the study are included in the article/supplementary materials, further inquiries can be directed to the corresponding authors.
